# Oncologic outcome with versus without target volume compartmentalization in postoperative radiotherapy for oral cavity squamous cell carcinoma

**DOI:** 10.3389/fonc.2024.1362025

**Published:** 2024-03-25

**Authors:** Elena Riggenbach, Manuel Waser, Simon A. Mueller, Daniel M. Aebersold, Roland Giger, Olgun Elicin

**Affiliations:** ^1^ Department of Radiation Oncology, Inselspital, Bern University Hospital, University of Bern, Bern, Switzerland; ^2^ Department of Otorhinolaryngology, Head and Neck Surgery, Inselspital, Bern University Hospital, University of Bern, Bern, Switzerland; ^3^ Department of Otorhinolaryngology Head and Neck Surgery, University Hospital and University of Zurich, Zurich, Switzerland

**Keywords:** head and neck cancer, oral cavity cancer, head and neck squamous cell carcinoma, postoperative radiotherapy, de-escalation, compartmentalization, radiotherapy, head and neck surgery

## Abstract

**Background and purpose:**

The volume treated with postoperative radiation therapy (PORT) in patients with oral cavity squamous cell carcinoma (OCSCC) is a mediator of toxicity affecting quality of life. Current guidelines only allow for very limited reduction of PORT volumes. This study investigated the safety and efficacy of de-intensified PORT for patients with OCSCC by refined compartmentalization of the treatment volume.

**Materials and methods:**

This retrospective cohort study identified 103 OCSCC patients treated surgically from 2014 to 2019 with a loco-regional risk profile qualifying for PORT according to guidelines. PORT was administered only to the at-risk compartment and according to a refined compartmentalization concept (CC). Oncological outcome of this CC cohort was compared to a historical cohort (HC) of 98 patients treated before the CC was implemented.

**Results:**

Median follow-up time was 4.5 and 4.8 years in the CC and HC cohorts, respectively. In the CC cohort, a total of 72 of 103 patients (70%) had a pathological risk profile that allowed for further compartmentalization and, hence, received a reduced treatment volume or omission of PORT altogether. Loco-regional control at 3 and 5 years was 77% and 73% in the CC cohort versus 78% and 73% in the HC (*p* = 0.93), progression-free survival was 72% and 64% versus75% and 68% (*p* = 0.58), respectively. Similarly, no statistically significant difference was seen in other outcome measures.

**Conclusions:**

De-intensified PORT limiting the treatment volume to the at-risk compartment or avoiding PORT altogether for low-risk patients with OCSCC does not seem to compromise disease control in this retrospective comparison. Based on these hypothesis-generating findings, a prospective study is being planned.

## 1 Introduction

Oral cavity squamous cell carcinoma (OCSCC) represents one of the most frequently diagnosed head and neck malignancy. Despite advances in treatment strategies and technology, OCSCC remains a significant cause of morbidity and mortality (1). Primary surgery with or without postoperative radio(chemo)therapy or primary radio(chemo)therapy are treatment options for patients with OCSCC. While different approaches are effective, they incur long-term morbidity that escalates with treatment intensity (2, 3). Reducing target dose and volume in radiotherapy (RT) or omitting postoperative RT (PORT) altogether are important potential toxicity-mitigation strategies that may improve quality of life (4, 5). To maintain oncological outcomes while reducing the toxicity, an appropriate definition of candidates qualifying for RT volume reduction or even complete omission due to a lower risk of recurrence is essential.

Many factors influence survival and loco-regional tumor control in patients with head and neck cancers. The presence of remaining postoperative microscopic/macroscopic disease at the margins of resection (R1/R2) and/or the presence of extracapsular extension of nodal disease (ECE) in the neck have been clearly defined as poor prognostic features. In patients with these high-risk features, both postoperative radiation therapy (PORT) and additional concurrent chemotherapy (6–9) improve loco-regional control as well as overall survival (OS). The presence of other “minor” adverse risk factors, such as multiple positive lymph nodes (without ECE), perineural invasion (Pn1), vascular invasion (V1), lymphatic invasion (L1), pT3 or pT4 primary, and oral cavity primary cancers with positive lymph nodes in level IV or V, are generally established indications for PORT as well. The direct individual association of each of the minor risk factors on local, regional, or general outcome is, however, not clear.

**Figure 1:**
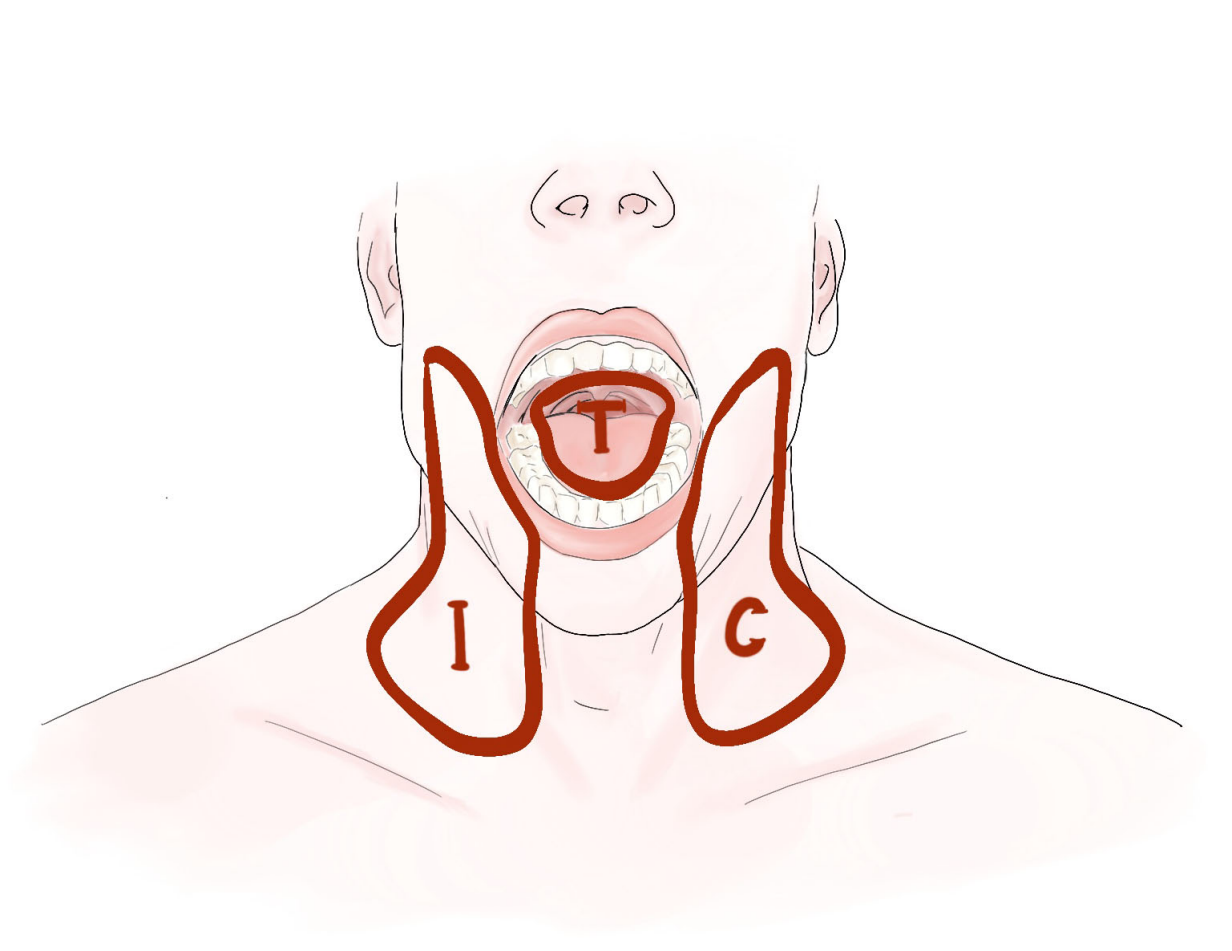
Illustrating the three compartments (T = tumor bed, I = ipsilateral neck, and C = contralateral neck) for postoperative radiotherapy in oral cavity squamous cell carcinoma.

There is no consensus to whether the primary tumor bed and each hemi-neck of the nodal basin should be considered as separate target compartments when these risk factors arise either only in the primary tumor bed or (hemi-)neck. According to current guidelines (10), the only accepted compartmentalization strategy in PORT for OCSCC is to spare the contralateral neck in case of a lateralized primary with local factor (R+ and/or >1 minor factor) and node negative disease after neck dissection. However, some aspects of PORT target volume definition are based on tradition, rather than evidence. Compared to the recommendations in the current guidelines, our institutional compartmentalization concept (CC) allows further de-intensification of PORT considering the tumor bed and each hemi-neck as three separate compartments for adjuvant RT decisions (**Figure 1**). Compared to the traditional holistic approach, the intent of the CC is to apply the required dose only to the compartment under risk.

The current study investigates further compartmentalization strategies in patients with operated OCSCC mandating PORT and compares their oncological outcome to a historical cohort (HC), where no compartmentalization was implemented.

## 2 Materials and methods

### 2.1 Study design and patient selection

A retrospective cohort study design was pursued following the Strengthening the Reporting of Observational Studies in Epidemiology (STROBE) guidelines (11). The study was approved by the regional ethics committee.

Eligible patients were 18 years of age or older with histologically confirmed diagnosis of OCSCC, treated with curatively intended primary surgery from January 2014 to March 2019. The study cohort was limited to patients having one of the following unfavorable loco-regional risk factors: close resection margin of histopathologically less than 5 mm from the tumor, perineural invasion, lympho-vascular space invasion, tumor (T-) stage ≥3, or more than one positive neck lymph node. PORT was administered according to our refined CC detailed below. Oncological outcome of this CC cohort was compared to a HC diagnosed and treated in our institution from January 2007 to December 2013. The period of the HC was started by the standard establishment of concomitant systemic treatment regimens, including cetuximab (12) (as an extrapolation from the definitive RT setting), which was similar to the period of treatment of the CC cohort. All patients in both cohorts were treated with intensity modulated RT techniques.

Patients with a previous head and neck squamous cell carcinoma (HNSCC) or previous RT to the head and neck area before the treatment course under investigation, or an active synchronous cancer at the start of treatment were excluded.

### 2.2 Treatment procedures and compartmentalization approach

**Figure 2:**
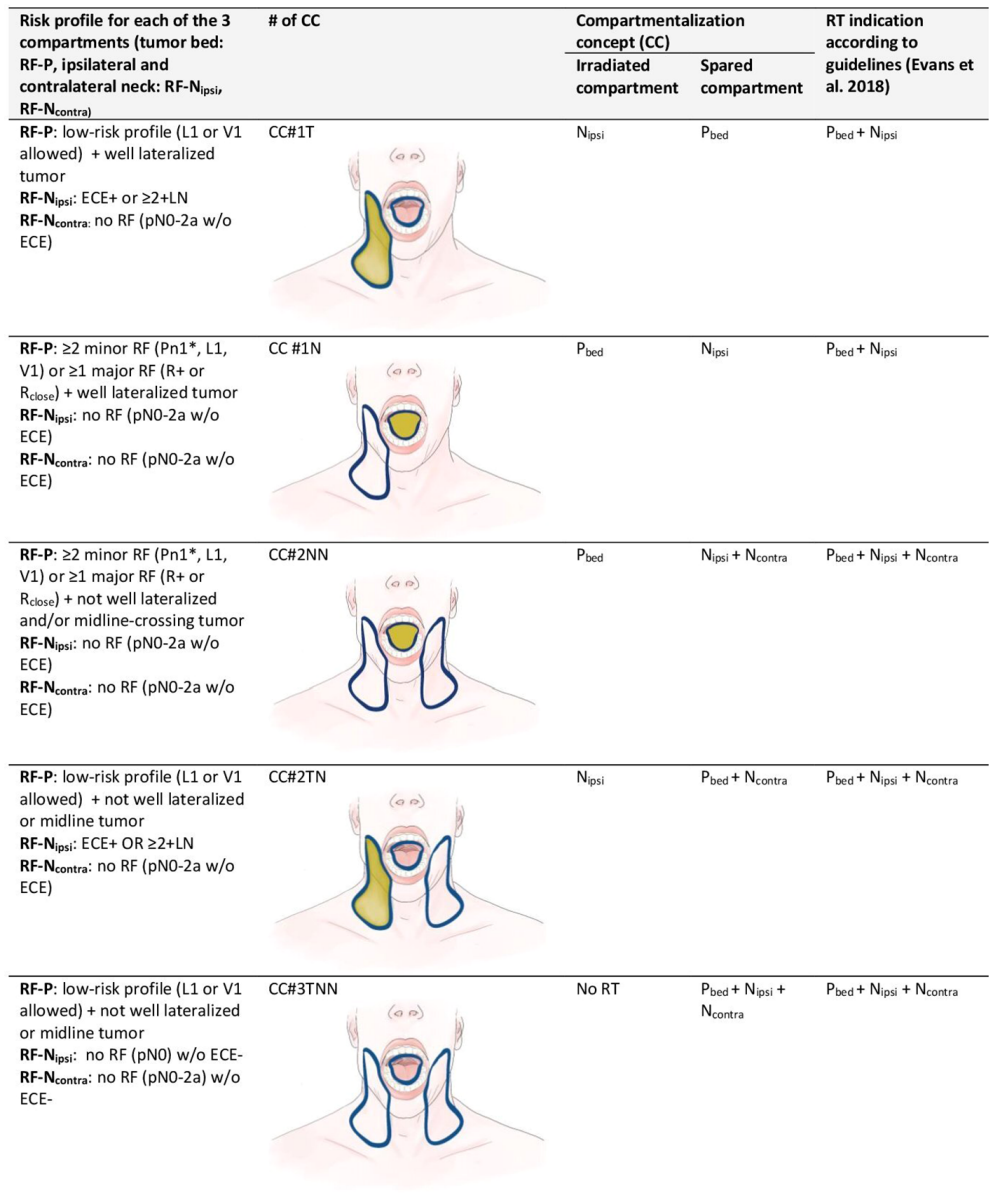
Compartmentalization concept (CC): clinical criteria and risk factors in each of the three compartments for one of five strategies of compartmentalization for postoperative radiotherapy in oral cavity squamous cell carcinoma. For staging the 7th edition of the International Union against Cancer (UICC), staging system was used. Each hemi-neck is regarded distinctly for pN-staging. Strategies of CC (#1–5) are shown as images (blue contour = compartment treated according to guidelines, yellow fields = compartments treated according to CC). CC = compartmentalization concept; L1 = lymphatic invasion; N_contra_ = compartment of the contralateral neck; N_ipsi_ = compartment of the ipsilateral neck; P_bed_ = compartment of the primary tumor bed; Pn1 = perineural invasion; R+ = positive resection margin; R_close_ = close resection margin (less than 5 mm from the tumor); risk factors (RF) of the primary tumor bed (RF-P), ipsilateral neck (RF-N_ipsi_) or contralateral neck (RF-N_contra_); V1= vascular invasion. *: exception Pn1 with a nerve diameter >0.1 mm or «named nerve» is enough as a sole factor.

Resection of the primary tumor was required with additional ipsi- or bilateral neck dissection according to multidisciplinary tumor board decision. All patients in the CC cohort met the criteria for PORT according to international consensus criteria (6, 10, 13). PORT was administered according to a clearly defined risk profile and only to the at-risk compartment (**Figure 2**). For RT of the primary tumor compartment we seek either an inadequate resection margin (R1/R2 or close) as a major risk factor or at least two minor risk factors (Pn1, L1, V1) with the exception of Pn1 with a nerve diameter >0.1 mm or “named nerve” that is enough as a sole factor warranting irradiation of the primary tumor bed (14, 15). We consider the operated pT3-4 tumor bed as pathological low-risk profile if no other risk factor is present (16, 17). To irradiate each hemi-neck, we seek either the presence of ECE or at least two involved lymph nodes. The theoretical treatment volumes (i.e., the compartments) according to the international consensus were recorded retrospectively for each patient depending on the pathological risk profile.

When treatment to the three compartments (i.e., tumor bed, ipsilateral and contralateral hemi-neck) according to CC was discordant from the guideline-conform PORT, it was classified into five categories: tumor bed spared (CC#1T), one hemi-neck spared (CC #1N), both hemi-necks spared (CC#2NN), tumor bed and one hemi-neck spared (CC#2TN), and all three compartments spared (CC#3TNN).

We detail our CC in **Figure 2** for various clinical settings and classify into five different variations of CC, depending on the compartment spared when comparing the treatment to the three compartments according to CC with the treatment corresponding scenarios governed by the pathologic risk profile. Patients in which the application of the refined CC did not result in a deviation from international consensus were classified as “no compartmentalization used”.

The concomitant systemic treatment was prescribed according to the international consensus guidelines, namely, based on the results of the EORTC 22931 (9) and RTOG 9501 (7), and Cetuximab (12) was used as substitute for cisplatinum-ineligible patients.

### 2.3 Diagnosis and follow-up

All treatment recommendations were discussed at the head and neck cancer specific multi-disciplinary tumor board after the initial histopathologic confirmation of OCSCC and again postoperatively, concerning the need of an adjuvant treatment. Our standard follow-up protocol is provided in **Supplementary Table S1**. The sequence and modalities of the diagnostic work-up were similar in the CC cohort and HC. In both cohorts, lymph node levels of the neck dissection were separated and individually marked during surgery before sending off to pathology. The number of positive (with and without ECE) and the total number of harvested lymph nodes were reported separately for each level by the pathologists. Staging for all patients was done according to the 7th edition of UICC (18).

### 2.4 Statistical analysis

The primary endpoint of this retrospective cohort study was to estimate the rate of loco-regional control (LRC), defined as the time from the date of histopathological diagnosis to the first documented local and/or regional recurrence. Median follow-up time was calculated by excluding the deceased patients.

Secondary endpoints included isolated local (LC), isolated regional (RC), LRC, and distant control (DMFS), progression-free survival (PFS) and OS. Kaplan–Meier method was used to depict survival curves for the oncologic endpoints, and the log-rank test for group comparisons. Analyses were performed using JMP^®^ statistical software (Version 16.2.0; SAS Institute Inc., Cary, North Carolina).

## 3 Results

### 3.1 Patient characteristics and treatment variables

The baseline demographic and clinical characteristics were comparable in both groups (**Table 1**). Of 187 consecutive patients with OCSCC treated with curative intent in our hospital from January 2014 to March 2019, 103 patients had a pathological risk profile based on which, an adjuvant treatment (i.e., PORT with or without concomitant systemic treatment) was indicated according to international guidelines.

**Table 1:** Patient characteristics of the study cohorts.

	Modern cohort (*n* = 103)	Historic cohort (*n* = 98)	*p*-value
	Percent (*n*) or median (range)	Percent (*n*) or median (range)	
**Age at diagnosis (y)**	62 (28–95)	60 (20–89)	0.06
**Sex**			0.29
Male	60.2% (62)	67.3% (66)	
Female	39.8% (41)	32.7% (32)	
**Primary tumor location within oral cavity**			
Tongue	49.5% (51)	NA	
Floor of the mouth	19.4% (20)	NA	
Alveolus and gingiva	12.3% (16)	NA	
Buccal mucosa	4.9% (5)	NA	
Hard palate	1.0% (1)	NA	
Unclear/multiple sites infiltrated	10.0% (10)	NA	
**Pathologic AJCC tumor stage (7th ed.)**			< 0.01
pT1	27.2% (28)	15.3% (15)	
pT2	39.8% (41)	41.8% (41)	
pT3	7.8% (8)	8.2% (8)	
pT4a	24.3% (25)	17.3% (17)	
pT4b	1.0% (1)	17.3% (17)	
**Pathologic AJCC nodal stage (7th ed.)**			< 0.01
pN0	35.0% (36)	43.9% (43)	
pN1	16.5% (17)	17.3% (17)	
pN2a	1.0% (1)	3.1% (3)	
pN2b	28.2% (29)	21.4% (21)	
pN2c	9.7% (10)	14.3% (14)	
pNX	9.7% (10)		
**Pathologic AJCC stage classification (7th ed.)**			< 0.01
Stage I	15.5% (16)	10.2% (10)	
Stage II	12.5% (13)	22.4% (22)	
Stage III	18.4% (19)	10.2% (10)	
Stage IVA	50.5% (52)	39.8% (39)	
Stage IVB	2.9% (3)	17.3% (17)	
**Presence of ENE in pN>0**	46.3% (31)	45.5% (25)	0.06
**Median number of LN involved**	1 (0–16)	1 (0–9)	0.12
**Adjuvant treatment**			< 0.01
No adjuvant treatment	24.3% (25)	0% (0)	
Radiotherapy	32.0% (33)	46.9% (49)	
Radiotherapy with systemic treatment	43.7% (45)	53.1% (52)	
Cisplatin* 100 mg/ m^2^ three-weekly	35.9% (37)	44.9% (44)	
Cetuximab* weekly	7.8% (8)	8.2% (8)	

AJCC, American Joint Commission on Cancer; ENE, extranodal extension; LN, Lymph node; RT, radiation therapy; NA, not available.

*Cisplatin concomitant 100 mg/m^2^ three-weekly, cetuximab 400 mg/m^2^ loading dose 1 week prior to radiotherapy and concomitant 250 mg/m^2^ weekly.

The CC cohort comprised these 103 patients. The median age was 62 years (range, 28–95) and 60.2% of patients were male. Median number of total harvested lymph nodes was 48 (range, 17–128). Thirty-six patients (35%) had a node positive disease. Node positive to harvested ratio was median 2% (range, 0%–28%). Treatment strategy was surgery alone (without adjuvant therapy) in 25 patients (24.3%), surgery followed by PORT in 33 patients (32.0%), and PORT with concomitant systemic treatment in 45 patients (43.7%). Low-risk volume in PORT received a median dose of 50 Gy (range, 50–54), intermediate-risk volume 60 Gy (range, 55–60) and high-risk volume 66 Gy (range, 2–68) in 2 Gy daily fractions.

All 98 patients from the HC received standard PORT according to guidelines, and no further compartmentalization strategy was implemented at that time. As with the treatment technique, RT doses in the HC for low-, intermediate-, and high-risk volume did not differ from the CC cohort. Due to the amount of missing data, detailed patient and tumor characteristics such as the anatomical subsites, smoking, and alcohol consumption as well as some pathologic factors (L1, V1, and Pn1) were not extracted.

### 3.2 Compartmentalization concept

Of the CC cohort, 52 patients (50.4%) would have had the theoretical indication for irradiation of the tumor bed and the unilateral neck, while 51 patients (49.5%) would have had the indication to irradiate all three compartments (tumor bed and bilateral neck) according to the standard protocol. Due to the implementation of CC, only 20 patients (19.4%) were effectively irradiated to all three compartments, while 23 patients (22.3%) received PORT to the tumor bed and unilateral neck, one patient (1.0%) to bilateral neck without the tumor bed, seven patients (6.8%) unilateral neck without the tumor bed, and 27 patients (26.2%) tumor bed only. In 25 patients (24.3%) with indication for PORT according to the standard protocol, PORT was omitted altogether under the CC.

Comparing the treatment and volume applied according to our CC with the guideline-conform indications, a total of 72 patients (69.9%) had a pathological risk profile that allowed further compartmentalization and hence received a reduced treatment volume. The category of compartmentalization of PORT applied in these 72 patients using the CC is shown in **Table 2**. In the remaining 31 patients (30.1%), no further compartmentalization was deemed safe, and they hence received a treatment volume identical to established guidelines.

**Table 2:** Compartmentalization strategy used in the CC (compartmentalization concept) cohort.

# of CC	Spared compartment*	# of spared compartment(s)	Percent (*n*)
1	Tumor bed	1	4.9% (5)
2	One hemi-neck	1	26.2% (27)
3	Both hemi-necks	2	11.7% (12)
4	Tumor bed and one hemi-neck	2	24.3% (25)
5	All three compartments	3	2.9% (3)
0	No compartmentalization used	0	30.1% (31)

*In total, PORT was omitted in a total of 25 (24.3%) patients through the compartmentalization strategy.

### 3.3 Oncologic outcome

**Figure 3:**
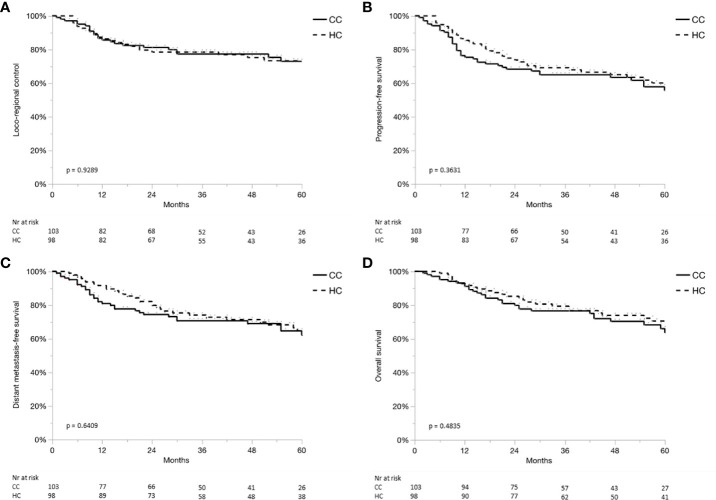
**(A–D)** Locoregional control **(A)**, progression-free survival **(B)**, distant metastases-free survival **(C)** and overall survival **(D)** for the compartmentalization cohort (CC, continuous line), and historical control (HC, dashed line).

Median follow-up time for patients still alive was 4.5 years (range, 0.3–7.4) for the CC cohort and 4.8 years (range, 0.2–8.9) for the HC. None of the oncological outcome measures showed a statistically significant difference when comparing survival curves of the CC cohort with the HC, that is, the null hypothesis of the log-rank test was retained for LC, RC, LRC, PFS, DMFS, and OS (**Table 3**). Detailed actuarial survival data are presented in **Figure 3**.

**Table 3:** Oncologic outcome with versus without target volume compartmentalization.

	CC cohort		Historical cohort		
Outcome	3-year (%)	5-year (%)	3-year (%)	5-year (%)	*p*-value
Local control	88.3	83.4	82.9	77.6	0.25
Regional control	87.6	87.6	85.8	83.8	0.64
Locoregional control	77.3	73.0	78.5	73.3	0.93
Progression-free survival	72.5	63.8	74.7	67.9	0.58
Distant metastasis-free survival	86.6	86.6	87.6	87.6	0.72
Overall survival	76.6	63.6	79.4	70.6	0.48

Isolated nodal failure occurred in four out of 67 patients (6%) from the CC cohort, where irradiation to at least one hemi-neck was spared. However, two of those four recurrences occurred in the irradiated volume. Local failures were observed in four out of 33 patients (12%) from the CC cohort, where irradiation of the tumor bed was spared.

## 4 Discussion

In this retrospective study of OCSCC with adverse features, the efficacy and safety of further compartmentalizing PORT was evaluated. Compared to a HC, our approach did not impact loco-regional disease control or survival rates, suggesting our CC to be safe. To our knowledge, this is the first analysis implementing different compartmentalization strategies at once for a relatively large OCSCC cohort.

The indication for PORT for OCSCC is based on the presence of major (ECE, close or positive margins) and minor (Pn1, V1, L1, pT≥3, pN≥2, and lymph node involvement in level IV or V) pathologic risk factors. There is no consensus to whether the primary tumor bed and each hemi-neck of the nodal basin should be considered as separate target compartments when these risk factors arise either only in the (hemi-)neck or in the primary tumor bed. The only accepted compartmentalization strategy in PORT for OCSCC according to current guidelines (10) is, to omit the contralateral neck in case of a lateralized primary with local risk factors (R+ and/or >1 minor factor) and node negative disease after neck dissection.

A de-escalation of PORT in our cohort of OCSCC was possible in 70% of the patients, either with reduction of the treatment volume by means of sparing the compartment(s) not harboring the corresponding risk factors (45.7%) or omitting PORT altogether (24.3%).

RT is associated with significant acute and long-term toxicities, primarily mediated by treatment volume and prescription dose. Thus, a reduction in radiation volume is expected to have a direct impact on acute and late toxicity as well as patients’ quality of life. This, in turn, would potentially allow for selective treatment intensification if deemed necessary. Toxicity mitigation by compartmentalization is to be weighed against the risk of loco-regional recurrence in untreated compartments, but our CC demonstrates this to be feasible without impacting the oncological outcome. Compared to the classical holistic approach, the CC also increases the rate of feasibility in re-irradiation scenarios in terms of the application of the adequate dose in required target volumes.

### 4.1 Omitting the pN0 and/or pN1 neck

The recently demonstrated long-term results of a prospective phase II study supports the safety of omitting the pathologically negative (pN0) neck (19). In this mixed cohort of operated HNSCC (*n* = 72 patients), including 14 patients with OCSCC, sparing the contralateral pN0 neck (74%) or even the ipsilateral pN0 neck (26%) resulted in an excellent unirradiated neck control of 97%. In our cohort, the omission of PORT to the hemi-neck was additionally allowed in the setting of a single-positive lymph node (pN1) without ECE. An earlier meta-analysis tested this approach but did not allow for general treatment recommendations due to large clinical heterogeneity of included studies (20). A more recent large retrospective study of patients with surgically treated OCSCC or oropharyngeal SCC shows that PORT to the pN1 neck in the absence of other adverse features might be associated with improved survival for pT2 disease or even pT1, especially in those younger than 70 years (21). While another, albeit smaller study only predicted a benefit for pN1 OCSCC if the lymph node yield at levels I–III was less than 20 (22). The hallmarks of a high-quality neck dissection, including at least 18 lymph nodes removed for levels I–III was again stressed in the recent ASCO guideline, making this a prerequisite for considering omission of the pN0 or pN1 neck in the PORT target volume (23). Although omission of RT to the pN1-neck without other adverse features seems to be currently accepted in most clinics, its controversy persists because PORT conferred a survival benefit in a recent large cancer registry-based study independent of adequacy of the neck dissection (24).

The lymph node yield in our CC cohort for patients receiving a neck dissection ensures the required quality. The abovementioned ASCO guideline (23) allows to omit PORT to the pN1 neck unless indications arising in the primary tumor, such as Pn1, L1, V1, or a pT≥3 primary are present. This prompts the question of whether in fact these local factors independently portend a higher risk for nodal recurrence. In a retrospective study on OCSCC, neither L1 nor V1 were independently associated with increased rates of regional or distant recurrence (25). A Japanese study group however revised their strategy of reducing treatment volumes when whole neck-PORT showed to be associated with a better OS, PFS, and LRC compared to limited-field-PORT in a retrospective analysis (26).

Isolated nodal failures in patients, where one or both hemi-necks were spared, was low (6%) in our cohort, suggesting a correct selection of patients with nodal low-risk profile (**Figure 2**) where irradiation of the nodal compartments might be safely omitted.

### 4.2 Omitting the primary tumor bed (CC#1T and CC#2TN)

Another compartmentalization strategy for HNSCC is the omission of the primary tumor bed for patients with a favorable local risk profile that receive PORT to the at-risk areas in the involved neck. Why the postoperative primary tumor bed should be irradiated in the presence of multiple nodal positivity and/or ECE, whereas the same tumor bed would not receive any radiation if the neck is pN0-1 lacks a logical rationale (27). A national patterns of care study revealed no consensus on this issue with 70% of the centers not separating the tumor bed from the dissected nodal levels, and 30% allowing for this type of de-escalation (27).

The recent prospective single-arm phase 2 “AVOID” trial for human papilloma virus-associated oropharyngeal SCC has explored de-intensification for PORT, which resulted in a 2-year local recurrence-free survival of about 98%. The investigators concluded this to be a safe strategy worthy of further study (28). However, one has to be aware that the incidental dose to the primary tumor bed in oropharyngeal SCC is somewhere between 30 Gy and 43 Gy (29) even if only the neck is targeted, which might be high enough to effectively sterilize residual microscopic disease, especially for HPV-associated oropharyngeal SCC. A more significant dose reduction however occurs in the oral cavity due to geometrical relationship and distance between the nodal and primary tumor bed volumes (30) resulting in a more evident compartmentalization effect when sparing the primary tumor bed for OCSCC. In addition to the anatomical, and as a result, dosimetric differences when considering the CC, OCSCC and oropharyngeal SCC (especially HPV+) are known to be distinct diseases from a biological perspective.

According to the recently published AIRO-GORTEC consensus for early stage OCSCC (15), omitting irradiation of the primary tumor bed should be investigated further for OCSCC, given the promising results observed in oropharyngeal SCC. With our current study we provide data that omission of the primary tumor bed may also be safe for selected OCSCC patients.

In terms of limitations, our cohort suffers from the intrinsic problems due to its retrospective nature. Potential unknown confounding bias cannot be eliminated. For the HC, data on pathological risk profile was incomplete and could therefore not be analyzed in the same detail as the CC cohort.

Additionally, owing to lack of randomization there are inherent differences between the two cohorts. Mitigating the impact of this limitation through statistical approaches such as propensity score matching would have been a futile effort with the available sample size and data.

Last, but not least, toxicity and quality of life data is not reported as it was largely missing and not recorded in the same systematic manner in the HC. However, the dose-volume and response (toxicity and quality of life) relationship is well-known, with smaller treatment volumes and lower doses being associated with less treatment-related toxicity in head and neck cancer (31–34). Justifying a de-escalation approach as a potential improvement in quality of life is only warranted as far as the non-inferiority of recurrence is ensured.

Therefore, there is a tremendous international effort for de-escalation of dose and target volumes in HPV-associated oropharyngeal cancer compared to the non-HPV-associated head and neck cancer (35).

Given the extent of missing data in the HC and minor known und potential unknown differences between the two cohorts, our exploratory comparative result should be considered as supplementary and is not the main emphasis of our paper.

## 5 Conclusions

With implementing a clearly defined strategy of further compartmentalization based on the pathologic risk profile in the respective compartment, a de-escalation of PORT is possible in the majority of OCSCC patients by reducing treatment volume or omitting PORT altogether. No compromise in disease control was seen when compared to a historical control. Based on these hypothesis-generating findings, a prospective trial is being designed.

## Data availability statement

The raw data supporting the conclusions of this article will be made available by the authors, without undue reservation.

## Ethics statement

The studies involving humans were approved by Regional ethics committee of Bern (2021-00616). The studies were conducted in accordance with the local legislation and institutional requirements. The participants provided their written informed consent to participate in this study.

## Author contributions

ER: Conceptualization, Data curation, Methodology, Project administration, Visualization, Writing – original draft, Writing – review & editing. MW: Data curation, Writing – original draft, Writing – review & editing. SM: Data curation, Methodology, Writing – original draft, Writing – review & editing. DA: Conceptualization, Methodology, Resources, Writing – review & editing. RG: Conceptualization, Data curation, Investigation, Resources, Supervision, Validation, Writing – review & editing. OE: Conceptualization, Formal analysis, Investigation, Methodology, Project administration, Supervision, Validation, Writing – review & editing.

## Supplementary material

Supplementary Table 1 (anonymized and non-anonymized version).

## References

[1] Bray F Ferlay J Soerjomataram I Siegel RL Torre LA Jemal A. Global cancer statistics 2018: GLOBOCAN estimates of incidence and mortality worldwide for 36 cancers in 185 countries. CA Cancer J Clin. (2018) 68:394–424. doi: 10.3322/caac.21492 30207593

[2] Rogers SN Scott J Chakrabati A Lowe D. The patients’ account of outcome following primary surgery for oral and oropharyngeal cancer using a “quality of life” questionnaire. Eur J Cancer Care (Engl). (2008) 17:182–8. doi: 10.1111/j.1365-2354.2007.00832.x 18302656

[3] Trotti A Pajak TF Gwede CK Paulus R Cooper J Forastiere A. TAME: development of a new method for summarising adverse events of cancer treatment by the Radiation Therapy Oncology Group. Lancet Oncol. (2007) 8:613–24. doi: 10.1016/S1470-2045(07)70144-4 17543584

[4] Kelly JR Husain ZA Burtness B. Treatment de-intensification strategies for head and neck cancer. Eur J Cancer. (2016) 68:125–33. doi: 10.1016/j.ejca.2016.09.006 PMC5734050 27755996

[5] Spencer CR Gay HA Haughey BH Nussenbaum B Adkins DR Wildes TM. Eliminating radiotherapy to the contralateral retropharyngeal and high level II lymph nodes in head and neck squamous cell carcinoma is safe and improves quality of life. Cancer. (2014) 120:3994–4002. doi: 10.1002/cncr.28938 25143048 PMC4257883

[6] Bernier J Cooper JS Pajak TF Van Glabbeke M Bourhis J Forastiere A. Defining risk levels in locally advanced head and neck cancers: A comparative analysis of concurrent postoperative radiation plus chemotherapy trials of the EORTC (#22931) and RTOG (#9501). Head Neck. (2005) 27:843–50. doi: 10.1002/hed.20279 16161069

[7] Cooper JS Pajak TF Forastiere AA Jacobs J Campbell BH Saxman SB. Postoperative concurrent radiotherapy and chemotherapy for high-risk squamous-cell carcinoma of the head and neck. N Engl J Med. (2004) 350:1937–44. doi: 10.1056/NEJMoa032646 15128893

[8] Huang DT Johnson CR Schmidt-Ullrich R Grimes M. Postoperative radiotherapy in head and neck carcinoma with extracapsular lymph node extension and/or positive resection margins: A comparative study. Int J Radiat Oncol Biol Phys. (1992) 23:737–42. doi: 10.1016/0360-3016(92)90646-Y 1618666

[9] Bernier J Domenge C Ozsahin M Matuszewska K Lefebvre JL Greiner RH. Postoperative irradiation with or without concomitant chemotherapy for locally advanced head and neck cancer. N Engl J Med. (2004) 350, 1945–52. doi: 10.1056/NEJMoa032641 15128894

[10] Evans M Beasley M. Target delineation for postoperative treatment of head and neck cancer. Oral Oncol. (2018) 86:288–95. doi: 10.1016/j.oraloncology.2018.08.011 30409314

[11] von Elm E Altman DG Egger M Pocock SJ Gøtzsche PC Vandenbroucke JP. The Strengthening the Reporting of Observational Studies in Epidemiology (STROBE) statement: guidelines for reporting observational studies. J Clin Epidemiol. (2008) 61:344–9. doi: 10.1016/j.jclinepi.2007.11.008 18313558

[12] Bonner JA Harari PM Giralt J Azarnia N Shin DM Cohen RB. Radiotherapy plus cetuximab for squamous-cell carcinoma of the head and neck. N Engl J Med. (2006) 354:567–78. doi: 10.1056/NEJMoa053422 16467544

[13] Haddad RI Hicks WL Hitchcock YJ Jimeno A Leizman D Pinto HA. NCCN Guidelines Version 1.2023 Head and Neck Cancers Continue NCCN Guidelines Panel Disclosures (2022). Available online at: https://www.nccn.org/home/member- (Accessed April 15, 2023).

[14] Bakst RL Glastonbury CM Parvathaneni U Katabi N Hu KS Yom SS. Perineural invasion and perineural tumor spread in head and neck cancer. Int J Radiat Oncol Biol Phys. (2019) 103:1109–24. doi: 10.1016/j.ijrobp.2018.12.009 30562546

[15] Merlotti A Alterio D Orlandi E Racadot S Bonomo P Franco P. AIRO GORTEC consensus on postoperative radiotherapy (PORT) in low-intermediate risk early stages oral squamous cell cancers (OSCC). Radiother Oncol. (2022) 177:95–104. doi: 10.1016/j.radonc.2022.10.035 36336113

[16] Fleury B Thariat J Barnoud R Buiret G Lebreton F Bancel B. Microscopic extensions of head and neck squamous cell carcinomas: Impact for CTV definition. Cancer/Radiotherapie. (2014) 18:666–71. doi: 10.1016/j.canrad.2014.04.006 24981411

[17] Yuen PW Lam KY Chan ACL Wei WI Lam LK. Clinicopathological analysis of local spread of carcinoma of the tongue. Am J Surg. (1998) 175:242–4. doi: 10.1016/S0002-9610(97)00282-1 9560130

[18] Sobin L Gospodarowicz M Wittekind C. AJCC Cancer Staging Manual. Oxford: Wiley-Blackwell (2009). doi: 10.1007/978-0-387-88441-7

[19] Contreras JA Spencer C DeWees T Haughey B Henke LE Chin RI. Eliminating postoperative radiation to the pathologically node-negative neck: Long-term results of a prospective phase II study. J Clin Oncol. (2019) 37:2548–55. doi: 10.1200/JCO.19.00186 31246526

[20] Moergel M Meurer P Ingel K Wendt TG Al-Nawas B. Effectiveness of postoperative radiotherapy in patients with small oral and oropharyngeal squamous cell carcinoma and concomitant ipsilateral singular cervical lymph node metastasis (pN1): A meta-analysis. Strahlentherapie und Onkol. (2011) 187:337–43. doi: 10.1007/s00066-011-2206-x 21603991

[21] Chen MM Harris JP Hara W Sirjani D Vasu Divi M. Association of postoperative radiotherapy. JAMAOtolaryngology–Head&Neck Surg. (2016) 142:1224–30. doi: 10.1001/jamaoto.2016.3519 27832255

[22] Feng Z Xu QS Qin LZ Li H Han Z. Predicting radiotherapy necessity in tongue cancer using lymph node yield. J Oral Maxillofac Surg. (2017) 75:1062–70. doi: 10.1016/j.joms.2016.10.005 27821247

[23] Koyfman SA Ismaila N Crook D D’Cruz A Rodriguez CP Sher DJ. Management of the neck in squamous cell carcinoma of the oral cavity and oropharynx: ASCO clinical practice guideline. J Clin Oncol. (2019) 37:1753–74. doi: 10.1200/JCO.18.01921 PMC7098829 30811281

[24] Suresh K Cramer JD. Postoperative radiation therapy vs observation for pN1 oral cavity squamous cell carcinoma. Head Neck. (2019) 41:4136–42. doi: 10.1002/hed.25958 31589006

[25] Adel M Kao HK Hsu CL Huang JJ Lee LY Huang Y. Evaluation of lymphatic and vascular invasion in relation to clinicopathological factors and treatment outcome in oral cavity squamous cell carcinoma. Med (United States). (2015) 94:1–7. doi: 10.1097/MD.0000000000001510 PMC4985367 26512553

[26] Makita C Kodaira T Daimon T Tachibana H Tomita N Koide Y. Comparisons of the clinical outcomes of different postoperative radiation strategies for treatment of head and neck squamous cell carcinoma. Jpn J Clin Oncol. (2017) 47:1141–50. doi: 10.1093/jjco/hyx137 29036621

[27] Elicin O Putora PM Siano M Broglie MA Simon C Zwahlen D. A review of controversial issues in the management of head and neck cancer: A swiss multidisciplinary and multi-institutional patterns of care study—part 2 (radiation oncology). Front Oncol. (2019) 9:1126. doi: 10.3389/fonc.2019.01126 31709186 PMC6822015

[28] Swisher-McClure S Lukens JN Aggarwal C Ahn P Basu D Bauml JM. A phase 2 trial of alternative volumes of oropharyngeal irradiation for de-intensification (AVOID): omission of the resected primary tumor bed after transoral robotic surgery for human papilloma virus–related squamous cell carcinoma of the oropharynx. Int J Radiat Oncol Biol Phys. (2020) 106:725–32. doi: 10.1016/j.ijrobp.2019.11.021 31785337

[29] Lazarev S Todorov B Tam J Gupta V Miles BA Lee N. Adjuvant radiation in the TORS era: Is there a benefit to omitting the tumor bed? Pract Radiat Oncol. (2017) 7:93–9. doi: 10.1016/j.prro.2016.08.002 28274400

[30] Cui T Ward MC Kittel JA Joshi N Koyfman SA Xia P. Dosimetric benefits of omitting primary tumor beds in postoperative radiotherapy after transoral robotic surgery using the auto-planning technique. Cureus. (2021) 13(9). doi: 10.7759/cureus.18065 PMC8520787 34671536

[31] Murphy BA Gilbert J Ridner SH. Systemic and global toxicities of head and neck treatment. Expert Rev Anticancer Ther. (2007) 7:1043–53. doi: 10.1586/14737140.7.7.1043 17627463

[32] Langendijk JA Doornaert P Verdonck-de Leeuw IM Leemans CR Aaronson NK Slotman BJ. Impact of late treatment-related toxicity on quality of life among patients with head and neck cancer treated with radiotherapy. J Clin Oncol. (2008) 26:3770–6. doi: 10.1200/JCO.2007.14.6647 18669465

[33] Van den Bosch L van der Schaaf A van der Laan HP Hoebers FJP Wijers OB van den Hoek JGM. Comprehensive toxicity risk profiling in radiation therapy for head and neck cancer: A new concept for individually optimised treatment. Radiother Oncol. (2021) 157:147–54. doi: 10.1016/j.radonc.2021.01.024 33545258

[34] Sher DJ Moon DH Vo D Wang J Chen L Dohopolski M. Efficacy and quality-of-life following involved nodal radiotherapy for head and neck squamous cell carcinoma: the INRT-AIR phase II clinical trial. Clin Cancer Res. (2023) 29:3284–91. doi: 10.1158/1078-0432.CCR-23-0334 37363993

[35] Petrelli F Luciani A Ghidini A Cherri S Gamba P Maddalo M. Treatment de-escalation for HPV+ oropharyngeal cancer: A systematic review and meta-analysis. Head Neck. (2022) 44:1255–66. doi: 10.1002/hed.27019 35238114

