# Supplementary material for: Oncologic outcome with versus without target volume compartmentalization in postoperative radiotherapy for oral cavity squamous cell carcinoma
Source: Front Oncol. 2024 Mar 25;14:1362025. doi: 10.3389/fonc.2024.1362025 (PMC10999524; doi:10.3389/fonc.2024.1362025)
Supplement: Supplementary Table 1 — (anonymized and non-anonymized version). [file DataSheet_1.pdf]

Bern University Hospital
Department of Otorhinolaryngology
Head and Neck Surgery
Division Head and Chair:
Prof. Dr. med. Marco Caversaccio
Prof. Dr. med. Roland Giger

Adapted and translated from the original version (Prof. R. Giger)

Follow-up Protocol for Head and Neck Cancers (University Hospital, Department Head and Neck, Radiation Oncology, CMFS)

Year	1								2					3				4			5			≥ 6
Months *(after end of treatment)*	*1*	*2*	*3*	*4*	*5*	*6*	*9*	*12*	*13*	*15*	*18*	*21*	*24*	*25*	*28*	*32*	*36*	*37*	*42*	*48*	*49*	*54*	*60*	*1x/year*
**H&P exam** Treatment: RT / chemoRT	Individual (RO)	RO	RO @	RO		ENT	ENT	ENT	RO	ENT	ENT	ENT	ENT	RO	ENT	ENT	ENT	RO	ENT	ENT	RO	ENT	ENT	**Follow-up at referring doctor**
Treatment: Surgery alone	Individual (ENT)	ENT	ENT	ENT		ENT	ENT	ENT		ENT	ENT	ENT	ENT		ENT	ENT	ENT		ENT	ENT		ENT	ENT	**Follow-up at referring doctor**
**Imaging studies** -MRI (or CT) Head and Neck [a]			RO / ENT					ENT	RO				ENT	RO										
-PET/CT (if T3, T4 and/ or N+) (Restaging)				RO / ENT																				
-Sonography Neck (if T3, T4 and/or N+)						ENT					ENT													
-CT chest with contrast agent								ENT	RO				ENT	RO										
-Low-dose CT chest [b]																	ENT			ENT			ENT	ENT **1x/year**
**Evaluationof Oral Cavity**	CMFS				CMFS				CMFS				CMFS	CMFS	CMFS	CMFS	CMFS	CMFS	CMFS	CMFS 🦷	CMFS	CMFS	CMFS	CMFS
**Psychooncological assessment**								Psycho-Oncology	Psycho-Oncology	Psycho-Oncology														
TSH [c]						RO			RO					RO										
EBV monitoring [d]				RO					RO					RO										

Legend: **Radiation Oncology** (RO) | **ENT** | **CMFS** | **Psycho-Oncology**

The department, which treated the patient last is responsible for organization of the follow-up!

[a] Imaging Face and Neck as initial imaging for all carcinoma (exception: stage-dependant individual imaging for Basalioma)
[b] low-dose CT chest without contrast in active smokers or ex-smokers (<15 years) / Pulmonary fibrosis / High-risk carcinoma of the salivary gland and age <81 years
[c] if neck irradiated.
[d] EBV-DNA-PCR plasma level in patients with nasopharyngeal cancer (comparison with baseline-value; if initially determined and >0)
@ Referral mail to: orl-tumorboard@insel.ch
🦷: Further follow-up according to individual decisions by the department of CMFS (cranio-maxillofacial surgery) >> referral back to general dentist
